# Adaptation of social and non-social cues to direction in adults with autism spectrum disorder and neurotypical adults with autistic traits

**DOI:** 10.1016/j.dcn.2017.05.001

**Published:** 2017-05-17

**Authors:** Rebecca P. Lawson, Jessica Aylward, Jonathan P. Roiser, Geraint Rees

**Affiliations:** a Wellcome Trust Centre for Neuroimaging, University College London, 12 Queen Square London, WC1N 3BG, United Kingdom; b Institute of Cognitive Neuroscience, University College London, 17 Queen Square, WC1N 3AZ, United Kingdom

**Keywords:** Social cues, Directional cues, Adaptation, Autism, Autistic traits, Sensory processing

## Abstract

Perceptual constancy strongly relies on adaptive gain control mechanisms, which shift perception as a function of recent sensory history. Here we examined the extent to which individual differences in magnitude of adaptation aftereffects for social and non-social directional cues are related to autistic traits and sensory sensitivity in healthy participants (Experiment 1); and also whether adaptation for social and non-social directional cues is differentially impacted in adults with Autism Spectrum Disorder (ASD) relative to neurotypical (NT) controls (Experiment 2). In Experiment 1, individuals with lower susceptibility to adaptation aftereffects, i.e. more ‘veridical’ perception, showed higher levels of autistic traits across social and non-social stimuli. Furthermore, adaptation aftereffects were predictive of sensory sensitivity. In Experiment 2, only adaptation to eye-gaze was diminished in adults with ASD, and this was related to difficulties categorizing eye-gaze direction at baseline. Autism Diagnostic Observation Schedule (ADOS) scores negatively predicted lower adaptation for social (head and eye-gaze direction) but not non-social (chair) stimuli. These results suggest that the relationship between adaptation and the broad socio-cognitive processing style captured by ‘autistic traits’ may be relatively domain-general, but in adults with ASD diminished adaptation is only apparent where processing is most severely impacted, such as the perception of social attention cues.

## Highlights

• Autistic traits negatively predict adaptation magnitude for social and non-social cues.
• Only adaptation magnitude for social eye-gaze is diminished in adults with ASD.
• High ADOS scores predict smaller aftereffects for head and eye-gaze direction.
• Diminished adaptation in autistic adults may only affect impaired perceptual domains.

## 1 Introduction

Autism Spectrum Disorder (ASD) is a neurodevelopmental condition characterised by social-communication difficulties and rigid or repetitive behaviour and restricted interests (American Psychiatric Association, 2013). There are a wide range of clinical phenotypes in ASD and it has been proposed that a wider continuum of individual differences in social-cognitive ability extends into the typical population and can be indexed by inter-individual differences in measures of autistic traits (Baron-Cohen et al., 2001a, Baron-Cohen et al., 2001b, Frith, 1991). Beyond the profound social-communication problems that are often characteristic of ASD the condition is also associated with a range of non-social symptoms such as hypersensitivity and hyposensitivity to perceptual stimuli, which now form part of the diagnostic criteria (American Psychiatric Association, 2013). Accordingly there is great interest in how social stimuli, such as eye-gaze, are processed in ASD (Nation and Penny, 2008), but there is also a growing body of work examining basic non-social visual processing (Simmons et al., 2009). However, perception of both social and non-social visual cues is influenced by mechanisms that produce experience-dependent modulation of visual sensitivity; known as adaptation (Webster, 2011). This mechanism warrants further study if we wish to understand commonalities, and differences, between basic sensory and social aspects of visual processing in ASD.

Adaptation is a central characteristic of neural systems, and can be defined as the short-term reduction in the responsiveness or sensitivity of neurons following prolonged exposure to a specific stimulus (or attribute) to which they are sensitive. The effects of adaptation can be measured invasively with electrophysiological recordings (Heeger, 1992), non-invasively with neuroimaging (Grill-Spector et al., 2006) and also behaviourally, in the form of perceptual aftereffects (Webster, 2011). Most commonly, adaptation aftereffects take the form of a perceptual bias towards an image or property of the opposite type. While it may seem counterintuitive to think of non-veridical perceptual biases as advantageous, adaptation is largely regarded as a beneficial neural gain control mechanism that aids perception (Kohn, 2007). For example, light adaptation in the retina allows us to discriminate small luminance changes, even though light intensity varies over many orders of magnitude, by altering the sensitivity of cells in the retina to the prevailing sensory conditions (Purpura et al., 1990). Problems with the adaptive ability to use recent sensory context to inform current perception may provide a mechanistic explanation for the sensory difficulties reported in ASD and, crucially, offers a mechanism that impacts on the perception of both social and non-social stimuli.

The appealing idea that the sensory and social symptoms of autism could be related to a common neural mechanism has gained attention in recent years. There are reports of reduced adaptation to facial identity, biological motion and eye-gaze in children with ASD (Ewing et al., 2013a, Pellicano et al., 2007, Pellicano et al., 2013, van Boxtel et al., 2016) and also reports of reduced adaptation to non-social stimuli such as numerosity (Turi et al., 2015). However, only one study has compared adaptation to social and non-social stimuli in the same group of participants and, for children with autism, reduced identity aftereffects were only seen for upright faces whereas preserved adaptation was seen for inverted faces and cars (Ewing et al., 2013b). This suggests that diminished aftereffects may be more apparent for social, relative to non-social, stimuli.

In adults with ASD, two recent studies suggest that adaptation to non-social auditory stimuli is diminished (Lawson et al., 2015a, Turi et al., 2016). In contrast, adaptive processing may be intact for some social cues, specifically facial identity (Cook et al., 2014) and expression (Rutherford et al., 2012). Preserved adaptation in adults with ASD may indicate that previous reports of diminished adaptation in autistic children reflect delayed or atypical developmental trajectories for these visual processes (Cook et al., 2014) and as far as we are aware, no studies have examined adaptation to social and non-social stimuli in the same group of adult participants. Therefore, it is far from clear whether adaptive coding of social and non-social cues is diminished in ASD.

One classic visual aftereffect is the tilt aftereffect – where a vertical grating appears tilted more to the left (or right) following prolonged (prior) exposure to a rightward (or leftward) grating (Gibson and Radner, 1937). Manifestations of directional aftereffects in high-level vision have also been demonstrated for the directional features of complex non-social visual stimuli such as horizontally rotated car orientation (Fang and He, 2005) but also for social attention cues like eye-gaze, head and body direction (Jenkins et al., 2006, Lawson et al., 2009, Lawson et al., 2011, Lawson and Calder, 2015). From the initial processing of these social attention cues we perceive where other people are attending, allowing us to make theory of mind judgements about the intentions, desires, and dispositions of others (Langton et al., 2000). Problems with the adaptive coding of directional cues may then have profound impacts on metalizing abilities, one of the core cognitive difficulties seen in ASD (Chung et al., 2014, Frith, 2001). Thus, ‘direction’ (or ‘orientation’) is an ideal stimulus attribute, common to both social and non-social stimuli and requiring similar processing demands, to test adaptation of social and non-social cues so that the interaction between stimulus category and group can be tested.

Here we present the results of two studies. In Experiment 1 we first we examine whether autistic traits negatively predict adaptation magnitude for two different types of social attention cue (eye-gaze direction, head direction) and a non-social directional cue signalled by a similarly complex visual stimuli (chair direction). We hypothesised that if the diminished aftereffects seen in children for social (Ewing et al., 2013a, Pellicano et al., 2007, Pellicano et al., 2013) and non-social (Turi et al., 2015) stimuli extend to the broad autism spectrum in the wider population, then there would be a negative correlation between autistic traits and adaptation magnitude. We also tested whether adaptation magnitude for social and non-social stimuli is related to individual differences in sensory sensitivity. We hypothesised that if sensory sensitivity, or ‘sensory overload’ (Ben-Sasson et al., 2009, Crane et al., 2009), is due to compromised adaptive processes in perceptual domains, then greater self-reported sensory sensitivity in the general population should be predictive of reduced adaptation magnitude. However, since previous studies comparing social and non-social adaptation in autistic children indicate that aftereffects for social-stimuli are diminished to a greater extent (Ewing et al., 2013b), we hypothesise that the negative relationship between adaptation magnitude and symptom severity will be stronger for social relative to non-social cues.

In Experiment 2, we examine adaptation to these same three directional cues (eye-gaze, heads and chairs) in adults with ASD and matched neurotypical (NT) control participants. The primary aim was to assess whether adaptation for social and non-social directional cues is diminished in adults with ASD and, if so, whether social and non-social stimuli are affected to the same extent. We also tested whether adaptation magnitude was negatively predictive of sensory/social symptoms.

Although many studies investigating adaptation to directional stimuli are concerned with direction-specific effects (e.g. adapting to leftward stimuli shifts perception away from left whereas adapting to rightward stimuli shifts perception away from right) adapting to left *and* right oriented stimuli simultaneously produces the net effect of increasing the number of “direct” responses to subsequently seen left and right facing stimuli. Models of pooled cell responses and empirical data support such effects of adaptation (Calder et al., 2008, Lawson et al., 2009, Lawson et al., 2011). So called “simultaneous adaptation”, has previously been demonstrated for a range of complex visual stimuli such as eye-gaze, body direction and head direction (Calder et al., 2008, Lawson et al., 2009, Lawson et al., 2011). As we do not wish to test the direction-specific effects of adaptation, but rather, differences in net adaptation magnitude across tasks (and between groups), we opted for a “simultaneous adaption” paradigm in both experiments reported in this manuscript.

## 2 Methods

### 2.1 Participants

In Experiment 1, twenty-eight healthy adult volunteers (16 male; aged 18–35 years; mean age 24.43; SD 3.52) with normal or corrected-to-normal vision took part. Subjects underwent screening for psychiatric and neurological disorders, and neither subjects nor their first degree relatives had previously received a clinical diagnosis of ASD in line with DSM-IV criteria (American Psychiatric Association, 2000). All subjects received monetary compensation for their time and travel expenses. Ethical approval was obtained from the Division of Psychology and Language Sciences/Institute of Cognitive Neuroscience Ethics Committee for Non-invasive Research on healthy adults (project identification number: JR/PWB/14-2-12a).

In Experiment 2, twenty participants with ASD were recruited via the Developmental and Executive Function database held at the UCL Institute of Cognitive Neuroscience. Participants had previously been diagnosed by an independent clinician, according to the DSM-IV (American Psychiatric Association, 2000) criteria. The Autism Diagnostic Observation Schedule (2nd edition) assessment (Lord et al., 2000) was completed by a qualified administrator to assess symptom severity in the ASD participants. Twenty NT participants with no previous or current psychiatric diagnosis served as controls. Two adults with ASD were excluded as they did not complete all three adaptation tasks. One additional ASD participant and one NT participant were removed due to incorrect button presses being logged throughout. This left nineteen NT participants and seventeen ASD participants in the final analysis [15 Asperger’s, 1 Autism Spectrum Disorder, 1 High Functioning Autism]. Demographic information can be found in Table 1. The Wechsler Adult Intelligence Scale (WAIS 3rd edition) had previously been administered to assess IQ. The ASD group were well matched with the NT group on both age, sex, and IQ (Table 1). Unfortunately, ADOS scores were not available for 4/17 of the ASD participants. All subjects received monetary compensation for their time and travel expenses. Ethical approval was provided by UCL Graduate School Ethics committee (4357/001).

**Table 1:** Participant demographics. The participants were matched on both age, sex, and IQ. ASQ, adult sensory questionnaire; ADOS, autism diagnostic observation schedule; C, communication; SI, social interaction; IQ, intelligence quotient.

	Group ASD		NT				
	mean(sd)	range	mean(sd)	range	*t*	*df*	*P*
Age(years)	42.9(11.8)	29–60	39.3(11.1)	23–60	0.934	34	0.357
Full Scale IQ	115.4(16.4)	80–136	111.7(13.2)	82–127	0.743	34	0.463
ASQ	13.9(4.4)	7–19	7.7(3.7)	2–14	4.469	34	<0.001
ADOS-total	9.0(2.9)	4–17					
ADOS-C	3.2(1.2)	1–6					
ADOS-SI	5.7(1.9)	3–11					

	Count		Count		*Chi-Sq*	*df*	*P*
Sex	13 males		10 males		2.21	1	0.137

### 2.2 Materials

The computer based tasks were run using Matlab 7.7.0471 (R2008b) (http://www.mathworks.co.uk/) and Cogent 2000 (http://www.vislab.ucl.ac.uk/cogent_2000).

Tasks were executed on a Dell Precision M4500 Laptop and presented on a 39.6 cm (15.6“) HD UltraSharp LED Display (1366 × 768 resolution). Participants viewed the stimuli at a distance of 57 cm with their head positioned in a chin rest to ensure that the images subtended the same visual angle in all participants, and direct eye-gaze fell at the level of the screen centre.

### 2.3 Procedure

**Fig. 1:**
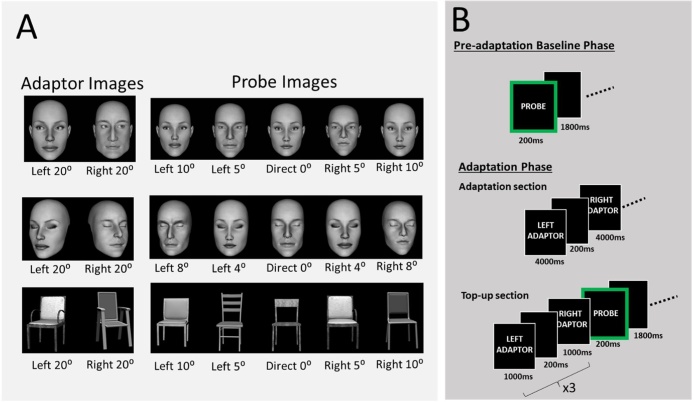
Sample stimuli, trial format, and procedure for the different adaptation tasks. (A) Examples of the social (eye-gaze, head direction) and non-social (chair orientation) stimuli used in the three tasks. (B) All three tasks had the same format comprising (i) a pre-adaptation baseline phase and (ii) an adaptation phase. An additional post-adaptation baseline phase also takes place but these data are not analysed. In the baseline phases participants categorised the direction (left, direct, or right) of probe images (green border) oriented in one of five directions. The adaptation phase consisted of two sections. In the adaptation section participants adapted to an alternating series of 20° left and 20° right oriented adaptors and in Section 2 the baseline phase was repeated with every probe image preceded by six top-up adaptor images. The primary outcome measure of adaptation magnitude is the change in the percentage of ‘direct’ responses made to the averted probe images in the adaptation phase relative to the pre-adaptation baseline. Participants completed these three phases three times, once for each stimulus type (eye-gaze, heads, and chairs) in a counterbalanced order for both experiments.

For all participants three separate adaptation tasks (eye-gaze direction, head direction and chair direction) were administered in a counter-balanced order. The procedure for each task was identical; accordingly, the general procedure will first be described, followed by specific details of the stimuli used in each task (Fig. 1A). Each adaptation task comprised two key phases: a pre-adaptation baseline phase (including practice), an adaptation phase which comprised two sections (Fig. 1B).

Pre-adaptation baseline phase: This comprised two identical blocks (practice and baseline) each showing probe stimuli across five orientations (40 stimuli in total). Each trial consisted of a probe image for 200 ms, and then a 1800 ms ISI. Participants categorised each probe image as facing ‘left’, “direct” or “right” with a button press. Presentation order was randomised.

Adaptation phase: The adaptation phase comprised two sections.

Section 1, ‘adaptation’: comprised a series of alternating left and right facing adaptor images presented for 4000 ms each (40 images in total). Adjacent adaptors never showed the same identity and a 200 ms ISI further served to eliminate any ‘apparent’ motion. Participants performed a dot detection task, which occurred on 10% of trials, to ensure attention throughout.

Section 2, ‘top-up’: contained the same probe images as the baseline block with exactly the same presentation times (200 ms each with a 1800 ms ISI for response logging). Again, participants categorised the direction as “left”, “direct”, or “right” however, preceding every probe images were six alternating left/right ‘top-up adaptors’ to maintain adaptation. Top-up images were presented for 1000 ms each, followed by a 200 ms ISI. In each trial top-up adaptors were always a different size and identity to the following probe stimuli.

Additionally a post-adaptation baseline phase took place after adaptation. This was identical to the pre-adaptation phase and provided a minimum standardised amount of time and intervening visual stimuli to allow the effects of adaptation to dissipate before the next adaptation task began. Previous studies in healthy volunteers indicate that the effects of eye-gaze adaptation are measurable on the discrimination of stimuli >300 s after adaptation has taken place (Kloth and Schweinberger, 2008). Measurements made in the post adaptation test phase are not used in the subsequent analysis.

### 2.4 Stimuli

Experimental stimuli were gray-scale computer generated images of faces and chairs created using DAZ 3D software (Daz productions, http://www.daz3d.com/). Examples of all three stimulus types can be seen in Fig. 1A.

Eye-gaze stimuli: Each probe eye-gaze image measured 10 cm (horizontally) by 6 cm (vertically) subtending a visual angle of 10° x 6°. Stimuli included five male and five female facial identities in which the eye-gaze was directed in one of five different orientations: 10° left, 5° left, 0° direct, 5° right and 10° right. These angles and image sizes have previously demonstrated significant eye-gaze adaptation aftereffects in a similar paradigm (Jenkins et al., 2006).

Head direction stimuli: Each probe head image measured 3.7 cm (horizontally) by 2.5 cm (vertically) subtending a visual angle of 3.7° × 2.5°. Stimuli included five male and female identities in which eyes were closed, and were presented at angles of either 8° left, 4° left, 0° direct, 4° right and 8° right. These angles and image sizes have previously demonstrated significant eye-gaze adaptation aftereffects in a similar paradigm (Lawson et al., 2011, Lawson and Calder, 2015).

Chair direction stimuli: Each probe chair image measured 3.1 cm (horizontally) and 4.7 cm (vertically) and subtended a visual angle of 3.1° x 4.7°. Ten different chair identities were used which were presented at angles of either 10° left, 5° left, 0° direct, 5° right and 10° right. These angles and image sizes have previously demonstrated significant chair direction adaptation aftereffects in a similar paradigm (Lawson and Calder, 2015).

Adaptor images: In each task adaptor images depicted 20° left and 20° facing exemplars of the task relevant stimuli (eye-gaze, head direction and chair direction accordingly). In all tasks these images were 25% larger than the corresponding probe images in order to rule out the possibility of adaptation effects being attributed to low level stimulus features.

### 2.5 Adaptation magnitude

The percentage of ‘direct’ responses to averted probe stimuli (collapsed across left and right) was calculated at baseline and at top-up (i.e. before and during adaptation). The extreme orientations, 8° (for heads) and 10° (for eyes and chairs) left and right, were included in this study as an “anchor” for perception of leftness and rightness only. Previous research (Calder et al., 2008, Jenkins et al., 2006, Lawson et al., 2011) indicates that these extreme orientations were subject to ceiling effects (i.e. rarely, or never, categorised incorrectly), and accordingly are not included in the analysis. The change in percentage of ‘direct’ responses to averted stimuli between the pre-adaptation baseline phase and the top-up phase was the critical outcome measure, indicating the overall magnitude of adaptation (i.e. adaptation magnitude = responses at top-up – responses at baseline).

### 2.6 Questionnaires

The Autism Quotient (AQ) (Baron-Cohen et al., 2001a, Baron-Cohen et al., 2001b) is a 50-item, self-report questionnaire designed in line with the DSM-IV-TR (American Psychiatric Association, 2000) to identify where an individual of normal intelligence lies on the autistic continuum by measuring their level of autistic traits. A score of 1 is attributed when a respondent rates an autistic-like behaviour as mild or strong. A respondent can score up to 50, with a score of ≥32 considered high. The AQ was administered to all participants in Experiment 1 to examine the relationship between adaptation magnitude and autistic traits in the normal population of neurotypical (NT) participants.

The Adult Sensory Questionnaire (ASQ) (Kinnealey et al., 1995) is a 26-item self-report, true-false questionnaire designed to identify sensory sensitivity in adults. True responses carry a point of 1 whereas false responses carry a point of 0. A score of 6 is considered average within healthy populations and a score of ≥10 is considered high. The ASQ provides a total score reflecting overall sensory sensitivity, i.e. inappropriate and exaggerated response to a typically harmless sensory stimuli. The ASQ was administered to all participants in Experiment 1 and Experiment 2 to measure sensory sensitivity.

### 2.7 Statistical analysis

Analysis was conducted using the Statistical Package for the Social Sciences, version 22 (SPSS Inc., Chicago, IL, USA). For each task, the main group effects of adaptation were analysed using repeated-measures 2 × 3 analysis of variance (ANOVA) with factors of: phase (baseline, top-up) and task (eye-gaze direction, head direction, chair direction). In Experiment 2 a between-subjects factor of group (ASD, NT) was added to the ANOVA. Independent samples *t*-tests were used to compare differences in adaptation magnitude where a significant interaction with group was identified in Experiment 2. All statistical tests are reported at a 2-tailed level of significance unless otherwise stated. Wherever the relationship between adaptation magnitudes was examined as an *a priori* hypothesised negative predictor of autistic traits/sensory sensitivity (Experiment 1) or ASD symptoms/sensory sensitivity (Experiment 2) bivariate correlations were conducted in line with our hypotheses (1-tailed). Steiger’s Z-test for correlated correlations was also used to investigate whether correlation coefficients for each task were statistically significantly different to one another (Steiger, 1980). Stieger’s Z test compares the equality of two correlation coefficients that share one variable in common while accounting for the correlation between the unshared variables.

## 3 Results

### 3.1 Experiment 1: adaptation and autistic traits

#### 3.1.1 Attention during the adaptation phase

Overall performance on the dot-probe detection task during the adaptation phase was ≥98.34% for all three tasks (Eye-gaze, M = 99.88, SD = 0.42; Head, M = 99.94, SD = 0.31; Chair, M = 99.94, SD = 0.31) suggesting that all participants were fixating on the adaptation stimuli and paying attention as instructed. To compare reaction time performance, the sample was median split into those scoring high (≥21) or low (≤20) on the AQ with reaction times compared via paired *t*-tests for each task. There was no significant difference in RTs for the high and low AQ groups for either task (eye-gaze t(13) = −0.460,P = 0.65, heads t(13) = −0.826, P = 0.42, chair t(13) = −0.194, P = 0.85), indicating equal attentional engagement for participants both low and high on the AQ for both social and non-social stimulus types.

#### 3.1.2 Effects of adaptation

First, to demonstrate that the three stimulus types were able to produce the expected effects of adaptation (e.g. an increase in ‘direct’ responses to averted stimuli in the top-up phase relative to baseline) we conducted a 2 × 3 repeated measures ANOVA comparing phase (baseline, top-up) and stimulus type (eye-gaze, head, chair). This demonstrated a significant main effect of phase (F(1,27) = 81.25, P < 0.001) indicating that exposure to adaptor images induced adaptation, a significant main effect of stimulus type (F(2,54) = 41.28, P < 0.001) and a significant stimulus type*phase interaction (F(2,54) = 14.25, P < 0.001) indicating that the effects of adaptation differed across the three tasks.

**Fig. 2:**
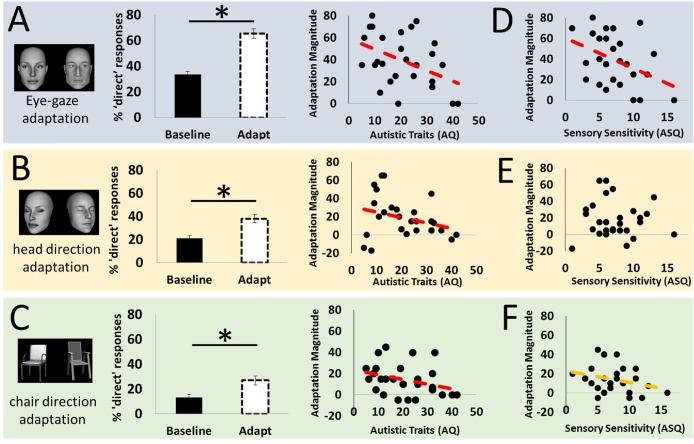
Experiment 1 results. (A–C) Adaptation aftereffects (an increase in ‘direct’ responses to averted stimuli following adaptation relative to the baseline phase) were measured for all three stimulus types (eye-gaze, heads and chairs) and correlated negatively with autistic traits in each case. (D–F) Adaptation magnitude only correlated negatively with sensory sensitivity for the eye-gaze and chair direction tasks. ^*^Denotes significance at P < 0.05. Significant correlations are marked with a dotted red trend line. Orange dotted line indicates trend significance.

Mean adaptation magnitude was 38.71% (SD = 24.01) for the eye-gaze task, 18.50% (SD = 21.86) for the head direction task and 14.00% (SD = 14.44) for the chair direction task. Echoing the main effect of adaptation in the above ANOVA paired *t*-tests demonstrate a significant increase in ‘direct’ responses to averted probes following adaptation for all three tasks (eye-gaze t(27) = 8.53, P < 0.001); head t(27) = 4.47, P< 0.001; chair t(27) = 5.12, P< 0.001; Fig. 2A–C).

#### 3.1.3 Relationship between adaptation magnitude and autistic traits

Adaptation magnitude correlated negatively with AQ score for the eye-gaze task (r(28) = −.44, P = 0.01; 1-tailed) and the chair orientation task (r(28) = −.33, P = 0.04; 1-tailed) indicating less adaptation in those with higher autistic traits (Fig. 2A & C). Although the correlation coefficient for the eye-gaze task and AQ score was numerically greater (in absolute terms) than the correlation coefficient for the chair task and AQ score, Steiger’s Z test revealed that these were not significantly different (Z = −.56, P = 0.28).

A negative correlation was also observed between adaptation magnitude for head direction and AQ score, but this narrowly missed statistical significance (r(28) = −.30, p = 0.064) although the direction of the relationship is consistent with the other two stimulus types (Fig. 2B). Additionally, Steiger’s Z tests revealed that the correlation coefficient between adaptation magnitude on the head direction task and AQ score was not significantly smaller than the corresponding correlation coefficients on either the eye-gaze (Z = −0.67, P = 0.24) or chair tasks (z = −.13, P = 0.44).

#### 3.1.4 Relationship between adaptation magnitude and sensory sensitivity

Adaptation magnitude for the eye-gaze task correlated negatively with sensory sensitivity as measured by the ASQ (r(28) = −.409, P = 0.015, 1-tailed; Fig. 2D), with a similar correlation that narrowly missed significance for the chair orientation task (r(28) = −.278, P = 0.076, 1-tailed; Fig. 2F). Adaptation magnitude for head direction did not correlate with sensory sensitivity (r(28) = −.05, P=.40, 1-tailed; Fig. 2E). The correlations between eye-gaze adaptation and ASQ, and chair orientation adaptation and ASQ were not significantly different from one another (Z = −0.66, P = 0.26). The correlation between eye-gaze adaptation and ASQ was, however, significantly greater than the correlation between head direction adaptation and ASQ (Z = −1.65, P = 0.04). The difference between the correlations with chair orientation and head direction adaptation narrowly missed significance (Z = −1.35, P = 0.08).

#### 3.1.5 Summary

Experiment 1 demonstrates that, at the group level, simultaneous adaptation to left and right oriented exemplars of social and non-social stimuli produce significant aftereffects (Fig. 2A–C). Additionally, we demonstrate that a negative relationship exists between susceptibility to adaptation aftereffects and autistic traits for both social and non-social stimuli. As such, those people who adapt less, or have more ‘veridical’ perception, also possess the greatest level of autistic traits. This finding is consistent with the idea that a continuum between health and disorder in the general population extends to a basic sensory feature of the autism spectrum. Furthermore, we demonstrate a negative relationship between magnitude of adaptation to direction of eye-gaze stimuli and sensory sensitivity which suggests a link between the sensory symptoms of ASD and adaptation of social stimuli. It remains to be seen, however, whether adaptation magnitude for social and non-social stimuli is actually diminished in ASD. We address this in Experiment 2.

### 3.2 Experiment 2: adaptation in adults with autism

#### 3.2.1 Attention during the adaptation phase

Across all participants performance on the dot-probe detection task during the adaptation phase was ≥98.52% for all three tasks (Eye-gaze ASD, M = 100%, SD = 2.02%; Eye-gaze NT, M = 97.36%, SD = 1.9%; Head ASD, M = 97.05%, SD = 1.3%; Head NT, M = 100%, SD = 1.3%; Chair ASD, M = 97.05%, SD = 2.01%; Chair NT, M = 100%, SD = 1.9%) suggesting that all participants were fixating on the adaptation stimuli and paying attention as instructed. An ANOVA investigating task (eye-gaze, head, chair) with a between subjects factor of group (ASD, NT) indicated no main effect of task (F(2,68) = 0.005, P = 0.99), group (F(1,34) = 0.53, P = 0.45), or interaction (F(2,68) = 1.59, P = 0.21).

An equivalent analysis conducted on RTs to detect the dots confirmed that there was no main effect of task (F(1.2,40.3) = 1.34, P = 0.27), group (F(1,34) = 1.95, P = 0.17), or interaction (F(2,68) = 1.98, P = 0.15), further indicating equal attentional engagement during the adaptation phase for both ASD and NT participants.

#### 3.2.2 Effects of adaptation

A 3 × 2 × 2 repeated measures ANOVA with factors of stimulus type (eye-gaze, head, chair), phase (baseline, top-up) and group (ASD, NT) was conducted on the percentage of direct responses made to averted stimuli. This revealed a main effect of stimulus type (F(2,68) = 39.43, P < 0.001) suggesting that there was a general difference in the percentage of direct responses made to each of the different stimulus types and a significant main effect of phase (F(1,34) = 193.52, P < 0.001) indicating that there was significant adaptation in general. A significant stimulus type*phase interaction indicates that the magnitude of adaptation differed significantly between the tasks (F(2,86) = 16.53, P < 0.001). The group*phase interaction was not significant (F(1, 34) = 0.08, P = 0.78) suggesting that adaptation magnitude did not differ between the groups overall. Importantly, however, there was a significant group*stimulus type*phase interaction (F(2,68) = 3.62, P = 0.03) indicating that the degree of adaptation to the different stimulus types differed between the groups.

**Fig. 3:**
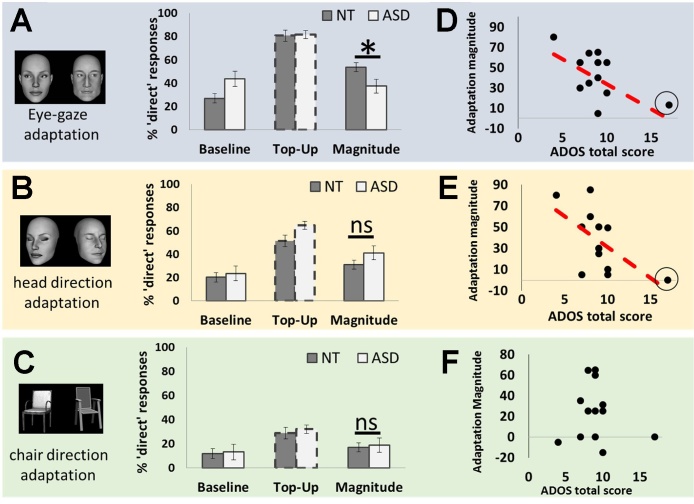
Experiment 2 results. (A–C) Adaptation aftereffects (an increase in ‘direct’ responses to averted stimuli following adaptation relative to the baseline phase) were measured for all three stimulus types (eye-gaze, heads and chairs) but the magnitude of this effect was only diminished in the ASD group for the eye-gaze stimuli. (D–F) In the ASD group, adaptation magnitude only correlated negatively autistic symptoms for the eye-gaze and head direction tasks. ^*^Denotes significance at P < 0.05. ns, not significant. Significant correlations are marked with a dotted red trend line.

Three 2 × 2 repeated measures ANOVAs with factors, phase (baseline, top-up) and group (ASD, NT) were conducted to investigate the simple interaction of phase*group for each stimulus type. As expected, the main effect of phase was significant for chair and head tasks, indicating that exposure to adaptor images caused adaptation (head: *F*(1, 34) = 76.75, *p <* 0.001); chair: *F*(1, 34) = 25.60, *P <* 0.001). The phase*group interaction was not significant for either task (chair: *F*(1,34) = 0.01, *P* = 0.80; head: *F*(1, 34) = 1.46, *P* *=* *0.*23) (Fig. 3B & C), suggesting no group differences in adaptation magnitude between the groups.

For the eye-gaze task, the main effect of phase was significant (*F*(1, 34) = 179.20, *P <* 0.001), again indicating successful adaptation. Importantly a significant group*phase interaction was detected (*F*(1,34) = 5.39, *P* = 0.026). This suggests that adults with autism have reduced eye-gaze adaptation magnitude, as confirmed with direct comparisons of adaptation magnitude (*t*(34) = −2.36, P = 0.024; mean ASD magnitude = 37% (SD = 24.5), mean NT magnitude = 53% (SD = 16.6)). Post-hoc *t*-tests revealed that at baseline, the ASD group was more likely to report averted gaze as direct (*t*(34) = 2.24, *P* = 0.032). The groups did not differ at top-up (*t*(34) = 0.15, *P* = 0.89). In other words, the ASD group was less accurate at categorising gaze direction at baseline, compared to the NT group, and therefore showed less susceptibility to adaptation (Fig. 3A).

To determine whether group differences in eye-gaze adaptation magnitude remained after accounting for general propensity to categorise stimuli as ‘direct’ we calculated an adaptation index (AI) as: AI = (responses at top-up − responses at baseline)/(responses at top-up + responses at baseline). Such an approach has been used previously in studies examining the relationship between fMRI adaptation and autistic traits (Ewbank et al., 2014). The groups significantly differed on this measure (t(34) = 2.16, P = 0.038) suggesting that, over and above general eye-gaze discrimination ability, adults with ASD show reduced eye-gaze adaptation.

#### 3.2.3 Relationship between adaptation magnitude and ADOS scores

In the ASD participants, eye-gaze adaptation magnitude was negatively predictive of the social-communication symptoms indexed by the ADOS (r(13) = −.515, P = 0.03, 1-tailed; Fig. 3D). When the outlier with highest ADOS score (marked with a circle on Fig. 3D; defined as >1.5 x the interquartile range) is removed from this analysis the correlation between eye-gaze adaptation and ADOS is slightly stronger (r(12) = −.532, P = 0.037, 1-tailed). Head direction adaptation also negatively correlated with ADOS scores, both with ((r(13) = −.598, P = 0.015, 1-tailed) and without (r(12) = −.527, P = 0.039, 1-tailed) the participant scoring highest on the ADOS included (Fig. 3E). There was no relationship, however, between chair direction adaptation and ADOS (r(13) = −.098, P = 0.375, 1-tailed; Fig. 3F). Steiger’s Z test indicated that the correlation between non-social (chair) adaptation magnitude and ADOS was lower than either of the corresponding correlations for social stimuli, though these narrowly missed significance (chairs vs. heads, Z = −1.63, P = 0.05; chairs vs. eye-gaze, Z = −1.29, P = 0.09).

#### 3.2.4 Relationship between adaptation magnitude and sensory sensitivity

A similar approach was taken to determine the relationship between eye-gaze, head and chair adaptation magnitude and sensory sensitivity as measured by the ASQ. Adaptation magnitude was not significantly related to ASQ for any stimulus type in the ASD group eye-gaze, r(17)=.32, P=.105; heads, r(17)=.073, P=.39; chairs, r(17) = −.04, P=.44, all 1-tailed). For the NT group sensory sensitivity was negatively correlated with non-social (chair) adaptation magnitude only (r(19) = −.421, P = 0.036, 1-tailed). Head and eye-gaze adaptation magnitude were not correlated with ASQ (head, r(19) = −.149, P=.21; eye-gaze, r(19)=.22, P=.18).

#### 3.2.5 Relationship between adaptation magnitude and AQ scores

In an exploratory analysis we examined the relationship between autistic traits and adaptation magnitude in the ASD and NT groups. Adaptation magnitude was not significantly related to autistic traits (AQ) for eye-gaze or chair stimuli in the ASD group (eye-gaze, r(17)=.27, P = 0.14, chairs, r(17) = −.05, P = 0.42, 1-tailed), but was negatively correlated with head direction adaptation (r(17) = −4.8, P = 0.026, 1-tailed). Adaptation magnitude was not significantly related to AQ for any stimulus type in the NT participants (eye-gaze, r(19)=.14, P = 0.29; chairs, r(19)=.07, P = 0.39; heads, r(19) = −.02, P = 0.47, all 1-tailed).

## 4 General discussion

### 4.1 Summary

We examined adaptation to both social and non-social visual cues in the general population (Experiment 1) and adults with ASD relative to NT participants (Experiment 2). We observed that autistic traits negatively predict adaptation magnitude for social (eye-gaze, head direction) and non-social (chair) directional cues in the hypothesised negative direction; where higher autistic traits are associated with reduced adaptation magnitude (Fig. 2A–C). However, only social eye-gaze adaptation was negatively related to sensory sensitivity. Additionally we report that adaptation magnitude is only diminished, at the group level, for social eye-gaze stimuli in adults with ASD and this is related, in part, to difficulties categorising eye-gaze direction at baseline (Fig. 3A). Adaptation for head and chair direction, however, is intact in ASD (Fig. 3B & C). Nonetheless, individual differences in social-communicative ability (ADOS scores) are negatively related to adaptation magnitude for both social-stimulus categories (eye-gaze and heads) in the ASD participants (Fig. 3D & E), whereas sensory sensitivity is not related to adaptation for any stimulus type.

### 4.2 Effects of attention

Simmons et al. (2009) suggest that reduced adaptation aftereffects reported in autistic children could be explained by reduced attention to or fixation on the adaptor images in the ASD group. As in recent studies (Ewing et al., 2013a) we addressed this explicitly in our design. Specifically we introduced short adaptation periods with emphasis on the importance of fixating during the procedure. Additionally, the number of ‘extreme’ (8°/10°) probe orientations included in each experiment was reduced relative to previous research which indicates perception of these non-ambiguous orientations, rarely changes following adaptation (Calder et al., 2008, Jenkins et al., 2006, Lawson et al., 2011). In doing so we reduced the overall length of each task to maintain attention. Crucially, however, the dot detection task in the adaptation phase of each task permitted us to implicitly measure attention to the adaptation stimuli, and we were able to demonstrate equal attentional engagement in the adaptation phase in both high and low-AQ participants in Experiment 1, and adults with ASD relative to NT adults in Experiment 2. Importantly, this suggests that reduced attention in the ASD group is not responsible for the reduced magnitude of adaptation found for the eye-gaze stimuli. It is interesting to note that considering we increased attention to the eye-gaze stimuli in this task, in a real world setting perhaps attenuated adaptation would be even more marked.

### 4.3 High vs. low-level adaptation

Adaptation is a canonical coding strategy echoed throughout the brain at different levels and on different timescales. In this study of high-level visual cues to direction, image identity and size changed between adaptor and probe images, effectively ruling out contributions from low-level retinotopic processes to these aftereffects. This is of relevance in light of previous studies in autistic adults showing intact facial identity adaptation using two different paradigms with more and less retinotopic dependence (Cook et al., 2014) and also one study in autistic children that suggests that adaptation conditioned upon retinotopic mechanisms is intact (Karaminis et al., 2015). Future studies should address if adults and children with ASD present with diminished or intact adaptation in low-level visual paradigms examining orientation (e.g. with gratings or gabor patches), though we predict that, at least in adults, adaptation for these very low-level image features is likely to be spared.

### 4.4 Adaptation in the non-clinical sample

Our findings in the non-clinical sample are consistent with reduced social and non-social adaptation in children with ASD (Ewing et al., 2013b, Pellicano et al., 2007, Pellicano et al., 2013, Turi et al., 2015) and also reduced fMRI adaptation to social and non-social visual stimuli as a function of autistic traits (Ewbank et al., 2014). However, the negative relationship between adaptation and autistic traits is not stronger for social, relative to non-social stimuli. This suggests that the association between adaptation and the broad socio-cognitive processing style indexed by the AQ may be relatively domain-general in the broader non-clinical population and underwrites the utility of the individual differences approach in understanding perceptual ability itself as a spectrum (Kanai and Rees, 2011). In Experiment 2 we do not observe this relationship between AQ and adaptation magnitude in the neurotypical sample, however in Experiment 1 every effort was made to recruit participants who scored over a wide range on the AQ questionnaire, increasing the sensitivity to pick up any relationship between autistic traits and task performance. This was not the case in Experiment 2 where we wished to compare two diagnostically distinct groups.

### 4.5 Relationship to sensory sensitivity

A failure of adaptive coding mechanisms, then has been proposed to offer a potential explanation for the sensory symptoms, known as sensory ‘overload’, that are prevalent in autism (Pellicano et al., 2007, Pellicano and Burr, 2012, Simmons et al., 2009). Somewhat consistent with this hypothesis we show that eye-gaze and chair adaptation magnitude, predicts trait measures of sensory sensitivity in our non-clinical sample. Specifically, those individuals who avoid sensory stimulation that other people would find innocuous (as measured by the ASQ) show the lowest susceptibility to adaptation aftereffects (Fig. 2D). Perhaps surprisingly, however, there was no relationship between adaptation magnitude and sensory sensitivity in our participants with ASD for any stimulus type. Since the stimuli employed here are all high-level visual cues to direction, future studies should explicitly examine the link between adaptation to low-level visual cues (e.g. light, dark, luminance, tilt etc.) as these may be more predictive of basic sensory sensitivity to lights, smells and touch. In support of this a recent study examining habituation to the loudness of simple auditory stimuli found that more complete adaptation was associated with reduced use of sensory avoidance strategies in ASD (Lawson et al., 2015a).

### 4.6 Are eyes special?

In Experiment 2 only eye-gaze adaptation was diminished in ASD at the group level (Fig. 3A), although reduced adaptation for both social stimulus types (eye-gaze and head direction) was associated with higher ADOS scores (Fig. 3D & E). Eye-gaze is a unique visual cue with a special role in establishing joint attention and signalling the intentions of other people (Emery, 2000), both abilities in which children and adults with ASD can show profound impairments (Baron-Cohen et al., 1985, Baron-Cohen et al., 2001a, Baron-Cohen et al., 2001b, Frith, 2001, Itier and Batty, 2009, Nation and Penny, 2008, Neumann et al., 2006, Pelphrey et al., 2005, Ristic et al., 2005, Senju et al., 2005, Senju and Johnson, 2009). Additionally, diminished adaptation aftereffects for social attention cues have been reported in healthy volunteers who do not believe the adaptor stimulus can “see” (Teufel et al., 2009), suggesting that the diminished eye-gaze adaptation we measure in adults with autism could be linked specifically to problems with representing the mental states of others (e.g. Theory of Mind (Baron-Cohen et al., 1985, Frith, 2001)).

The fact that the diminished eye-gaze adaptation reported here is related, in part, to poorer discrimination of eye-gaze at baseline supports the position that diminished adaptation emerges only for the most impaired domains of processing in adults with ASD and is consistent with the finding that eye-gaze adaptation in autistic children is also related to problems with categorising gaze direction (Pellicano et al., 2013). It is possible, then, that problems with adaptation may be more pervasive in children with ASD, affecting both social and non-social stimuli (Ewing et al., 2013a, Pellicano et al., 2007, Pellicano et al., 2013, Turi et al., 2015), but following a delayed or atypical developmental trajectory these difficulties resolve or improve for some domains of processing and remain for those where impairments are most severe. For example, face identity aftereffects may be intact (Cook et al., 2014), because adults with autism often present with face memory problems rather than face discrimination problems (Weigelt et al., 2012, Weigelt et al., 2013). This suggests that atypical adaptation may not be an enduring domain-general feature of ASD and should be considered in a broader developmental context as individuals with autism transition from children into adulthood. Ideally, future studies would examine adaptation in adults and children with ASD, using both social and non-social tasks.

### 4.7 Relationship to symptoms

Beyond the differences in adaptation reported at the group level it is also worth noting that reduced adaptation magnitude for both social stimulus types (heads and eye-gaze) was predictive of individual differences in social-communication symptoms in the ASD participants (Fig. 3D & E). We caution that since the ADOS was not administered to four of the ASD participants the sample size in these correlations is reduced, though we demonstrate that they are not driven by the identified outlier (see results). Interestingly, the ADOS assessment measures symptoms such as unusual eye-contact, poor social responses and limited reciprocal interaction (Lord et al., 2000); all of which would be expected to suffer in real-world social situations if an individual had problems updating representations of social attention cues (e.g. where people are looking). The present results suggest that this relationship is specific to the adaptation of social cues to direction, and not high level directional cues in general as there was no relationship between ADOS scores and chair adaptation magnitude. This is supported by recent study employing these same stimuli in healthy volunteers and found no cross-category adaptation for social and non-social directional cues (Lawson and Calder, 2015). This suggests that these aftereffects do not arise from abstract representations of ‘leftness’ or ‘rightness’ that affect any kind of stimulus, but rather from representations specific to each cue type.

### 4.8 Theoretical considerations

Computationally it has been proposed that adaptation can be modelled exclusively, or as a combination of, divisive normalization, gain control and using Bayesian generative models like predictive coding (Schwartz et al., 2007). Theoretically, it has recently been suggested that atypicalities in autistic perception could be explained under a Bayesian framework (Pellicano and Burr, 2012) or its neural instantiation, predictive coding (Friston et al., 2013, Lawson et al., 2014, Lawson et al., 2015b, Van de Cruys et al., 2014). For a comprehensive tutorial and review of recent Bayesian treatments of autism see Palmer et al., 2017. At the conceptual level, reduced aftereffects sit comfortably with the idea of reduced reliance on prior beliefs (Pellicano and Burr, 2012). However Bayesian computational models that allow the adaptor stimuli to affect the prior directly predict that perception should be pulled towards the adaptor and not away from it (Stocker and Simoncelli, 2006). Therefore, the link between adaptation and prior beliefs warrants further study and, at the level of explaining behavior, novel approaches may need to be considered. For example, it has recently been suggested that priors may be coded in the channel selectivity structure underlying the representation of different visual attributes, with adaptation reflecting short term changes in channel sensitivity (Clifford et al., 2015). In terms of eye gaze adaptation this has been captured computationally as a form of divisive normalization (Palmer & Clifford, 2017).

Predictive coding is the neural instantiation of Bayesian inference and predictive coding mechanisms have been shown to explain fMRI-adaptation to high-level visual stimuli (Ewbank et al., 2011). Reduced fMRI-adaptation to faces has been observed in ASD (Ewbank et al., 2016, Kleinhans et al., 2009, Swartz et al., 2013) and schizophrenia (Williams et al., 2013), suggesting a breakdown in predictive processing at the level of hierarchical neural computation. Furthermore, consistent with the findings of Experiment 1, reduced neural adaptation to faces, scenes and simple shapes has been shown in individuals high on measures of autistic traits (Ewbank et al., 2014). We would therefore hypothesize that fMRI adaptation studies investigating repetition suppression of social attention cues in ASD would echo the reduced behavioral effects of adaptation found here, at least for eye-gaze stimuli. These findings would be broadly consistent with the idea of a failure of sensory attenuation, or high expected precision on sensory inputs, in ASD (Friston et al., 2013, Lawson et al., 2014, Lawson et al., 2015b, Palmer et al., 2017).

### 4.9 Conclusions

These results advance our understanding of the links between the social and sensory features of ASD by examining a processing mechanism that impacts on both social and non-social stimuli and specifically addressing whether adaptation of these cues is differentially impaired. These results suggest that the relationship between adaptation and the broad socio-cognitive processing style encompassed by ‘autistic traits’ may be relatively domain-general similar to findings in autistic children, but in adults with ASD diminished adaptation is only apparent where processing is most severely impaired, such as perceiving social attention cues.

## Conflict of Interest

None.
